# Studies on prevalence of Hantavirus in small mammals in Southeast Asia: A systematic review and meta-analysis

**DOI:** 10.1371/journal.pntd.0014075

**Published:** 2026-03-12

**Authors:** Zixiao Guo, Hongxin Pan, Nini Wang, Yang Xiao, Qianwen Zhang, Changchun Ren, Puyu Liu, Qun Wu, Lijun Cai, Yang Cheng, Weixia Li, Dingwei Sun

**Affiliations:** 1 School of Public Health, Hainan Medical University, Haikou, Hainan, China; 2 School of Public Health, Shandong University, Jinan, Shandong, China; 3 Hainan Provincial Center for Disease Control and Prevention (Hainan Academy of Preventive Medicine), Haikou, Hainan, China; Linköping University: Linkopings universitet, SWEDEN

## Abstract

**Background:**

This study systematically analyzed the prevalence of Hantavirus (HVs) in small mammals across Southeast Asia to evaluate the risks of this zoonotic disease.

**Methods:**

We searched the Web of Science, PubMed, Embase, Scopus, and Cochrane Library for studies published up to 6 February 2025, extracted data from 28 eligible studies.

**Results:**

Meta-analysis revealed a pooled prevalence of HVs was 6.07% (986/11,806) in small mammals in Southeast Asia, with the highest prevalence in Indonesia (17.49%) and Singapore (10.53%). The prevalence was higher in samples detected by Enzyme-Linked Immunosorbent Assay (10.68%) and in rodents (8.07%).

**Conclusions:**

The results of our study highlight the urgency of strengthening surveillance in trade networks with Southeast Asia, particularly in high-risk areas such as Indonesia and Singapore, to mitigate the threat of imported zoonotic diseases.

## Author summary

Hantaviruses (HVs) are zoonotic pathogens that can cause severe diseases in humans, such as Hantavirus pulmonary syndrome (HPS) and Hemorrhagic Fever with Renal Syndrome (HFRS). Understanding the prevalence of HVs in small mammals is critical for assessing public health risks, especially in regions with high biodiversity and frequent international trade. In this study, we conducted a systematic review and meta-analysis of 28 studies to estimate the prevalence of HVs in small mammals across Southeast Asia. We found an overall prevalence of 6.07%, with higher rates in Indonesia and Singapore. Certain species, such as *Rattus norvegicus* showed particularly high infection rates. Detection methods also influenced prevalence estimates, with immunofluorescence assays reporting higher rates than molecular methods. Our findings underscore the need for enhanced surveillance in high-risk areas and trade networks to prevent the cross-border spread of Hantaviruses.

## Introduction

*Orthohantaviruses*, hereafter referred to as Hantaviruses (HVs), are negative-sense RNA viruses that reside within the subfamily *Mammantavirinae* of the family *Hantaviridae*, according to the most recent taxonomy from the International Committee on Taxonomy of Viruses (ICTV; https://ictv.global/) [1]. All human-pathogenic HVs belong to this genus, which includes the etiologic agents responsible for both Hemorrhagic Fever with Renal Syndrome (HFRS) and Hantavirus Pulmonary Syndrome (HPS) [2]. HV is a zoonotic pathogen transmitted by small mammals, which can cause HPS, a severe acute respiratory illness in the Americas primarily. The symptom is generally severe and associated with a mortality higher that 25% [3]. In the United States, the estimated case fatality rate is approximately 35% [4,5].The clinical features of HFRS are mainly fever, varying degrees of bleeding tendency, renal failure, headache, back pain, abdominal pain, and hypotension. Infection results from exposure to aerosols contaminated with HVs [6]. Annually, approximately 150,000–200,000 HFRS cases are reported worldwide, with a mortality as high as 15% [7,8]. HFRS is mainly prevalent in Asia and Europe, with China being the country most severely affected by the disease globally. Studies show that China accounts for more than 90% of all reported HFRS cases worldwide [9]. It is worth noting that since 2000, the incidence of HFRS in China has remained high, consistently ranking first in the world [6].

The primary vectors for HVs are small mammals, such as bats and rodents [10]. HVs have a wide range of hosts, which are large in number and often coexist with domestic animals or humans, thereby posing a significant risk of HVs infection to humans. Research indicates that vectors for HVs transmission include *Rattus norvegicus*, *Apodemus agrarius*, *Rattus flavipectus,* among others [11]. Therefore, monitoring the prevalence of small mammals for HVs is crucial for analyzing, assessing, and warning against HFRS. With global climate change and the deepening of China’s exchanges with the international community in transportation, trade, and tourism, pathogen-carrying organisms and the pathogens they carry can be introduced between international ports via vehicles, containers, goods, mail, etc., increasing the risk of imported diseases and potentially triggering domestic transmission [12]. Furthermore, studies have also postulated that HVs infections introduced through international freight channels via rodents may serve as a primary etiology [2].The frequent detection of rodents on incoming transport in China poses a direct risk of pathogen introduction, as evidenced by reports of HVs detection in these imported animals [13].

Hainan is located in the tropical region of China, which is favorable for the reproduction of small mammals. The rodent density exceeds 11.67% using fluorescence Polymerase Chain Reaction method [1]. According to the China Public Health Science Data Center, the number of HFRS cases in Hainan Province exhibited an increasing trend from 2014 to 2019 (https://www.phsciencedata.cn/Share). With the development of the Free Trade Port, the risk of imported HVs carried by small mammal hosts is higher, as small mammals can enter Hainan via ships, aircraft, etc. Current research is scattered across countries such as Vietnam, Thailand, and Indonesia, with no studies estimating the prevalence of HVs across the entire Southeast Asian region. Therefore, it is essential to identify HVs infection in small mammals and clarify the distribution of the virus in small mammals across Southeast Asia.

To understand the prevalence is critical for implementing effective control measures and facilitate the development of targeted preventive strategies for HVs, we conduct a comprehensive systematic review and meta-analysis to evaluate the prevalence of HVs in small mammals. Specifically, we explored the prevalence of HVs in small mammals across Southeast Asia, analyzing variations by geographic subregions, detection methods, host species, publication years, and sample sizes.

## Materials and methods

### Protocol and registration

The systematic review and meta-analysis were conducted based on the Preferred Reporting Items for Systematic and Meta-analysis (PRISMA) protocols [14] and prospectively registered on PROSPERO (CRD420251063857).

### Search strategy

A computerized search was performed in the Cochrane Library, PubMed, Embase, Web of Science, and Scopus databases for publicly available literature on surveys of HVs in small mammals in Southeast Asia. The search period covered the establishment of the databases up to 6 February 2025. The search terms were (Rodent OR Mouse OR Rats OR Mice OR Cricetid OR Bat) AND (Hantaviruses OR Hantaan virus OR Hemorrhagic fever with Renal Syndrome OR HFRS OR Hantavirus infection OR Hantavirus disease OR Hantavirus detection OR Hantavirus prevalence OR HPS OR Hantavirus Pulmonary Syndrome), with the region restricted to Southeast Asia.

### Literature inclusion and exclusion criteria

Inclusion criteria: (1) The study type is an observational study, including Cross-Sectional Studies (CSS); (2) Studies involving small mammals (bats, shrews, and rodents) carrying HVs; (3) Detection results must reflect natural infection (excluding experimental infections); (4) The research location in Southeast Asia, including Vietnam, Laos, Cambodia, Thailand, Myanmar, Malaysia, Brunei, Singapore, Indonesia, East Timor, and the Philippines.

Exclusion criteria: (1) Unable to extract or convert key parameters such as sample size, number of positives, or positive rate; (2) Interventional studies, reviews, case reports, conference abstracts, non-original studies, and duplicated publications.

### Literature screening and data extraction

Two reviewers carried out the extraction and recording of data from each chosen study independently. In case of any discrepancies between the reviewers or any ambiguity regarding the suitability of a study, additional reviewers were consulted to resolve the issue. Information was recorded as follows: First author, Year of publication, Country, Research subject type, Detection method, Total sample size, Number of HVs-positive cases, Study design type, and Study quality. Microsoft Excel 2021 was used for data management.

### Quality assessment

The quality assessment of included cross-sectional studies was conducted using the cross-sectional study evaluation criteria recommended by the Agency for Healthcare Research and Quality (AHRQ) [15]. Scores of 8–11, 4–7, and 0–3 represented high, fair, and poor quality, respectively. Two independent reviewers conducted the evaluations, and any disagreements resolved through discussion or by a third reviewer.

### Statistical analysis

We quantified the prevalence of HVs in small mammals in Southeast Asia through meta-analysis using R4.5.0. Study heterogeneity was evaluated by I² statistics to determine the selected model (random-effects for I² > 50%, otherwise fixed-effects). Publication bias was assessed by funnel plots, Egger’s regression test, Begg’s rank correlation tests [16]. Robustness was verified by sensitivity analysis excluding individual studies sequentially [17]. All effect estimates are reported as 95% confidence intervals (CI) and p < 0.05 defined statistical significance.

## Results

### Database search

**Fig 1:**
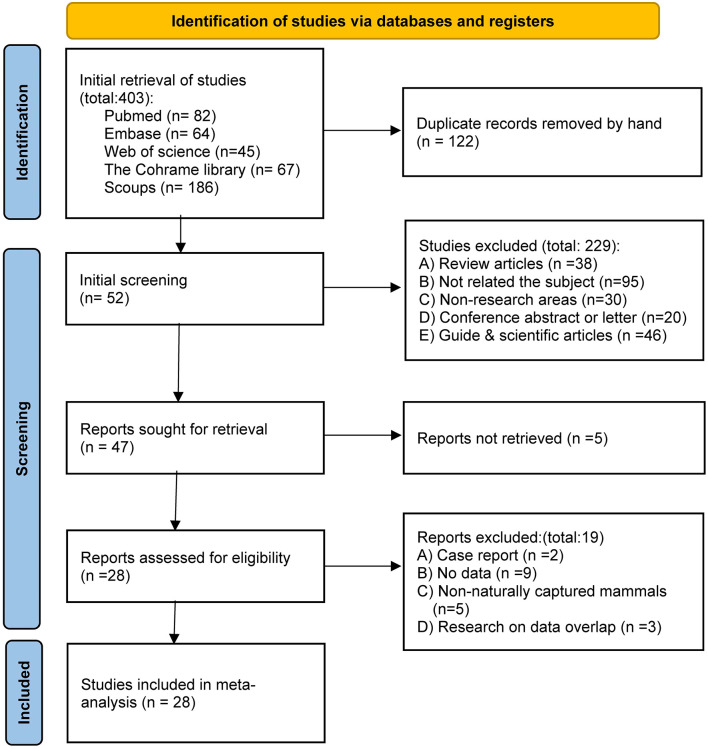
Flowchart illustrating the study selection process for the meta-analysis.

A total of 403 studies were identified from database searches. After removing 122 duplicates, 281 studies were screened. A total of 229 studies were excluded based on titles and abstracts. Full-text assessment was conducted for 47 studies (5 were not retrieved), of which 19 were excluded. Finally, 28 studies were included in the meta-analysis(Fig 1) [2,3,5,7, 18–41].

### Study characteristics

Ten studies (36%) were published after the year 2015, and 4 studies (14%) were published before 2000. All selected 28 studies (100%) were cross-sectional studies. Sample sizes varied widely, with the smallest sample reported being 53 individuals surveyed in Malaysia [34] and the largest sample being 1311 individuals surveyed in Vietnam [40]. Overall agreement for the rating of the quality of reporting and methodology between the two assessors was 93% (S1 Table). Twenty-two articles were high quality literature, and 6 articles were fair quality literature (S2 Table).

### Meta-analysis

#### Prevalence of HVs in small mammals in Southeast Asia.

**Fig 2:**
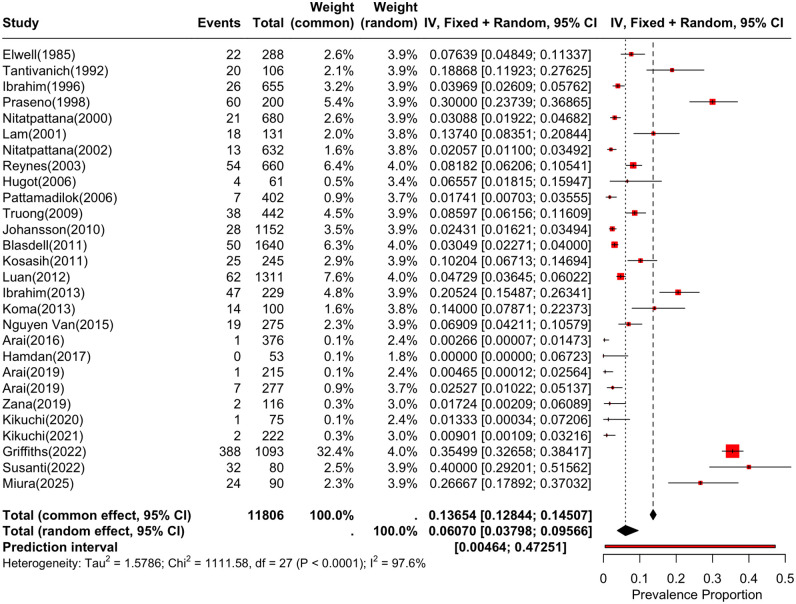
Forest map of the prevalence of Hantavirus in small mammals in Southeast Asia.

A random-effects model was used for the meta-analysis due to the high degree of heterogeneity in prevalence of HVs in small mammals in Southeast Asian (I² = 97.6%, *p* < 0.01). The results showed that the prevalence of HVs in small mammals in Southeast Asian was 6.07% (95%CI, 3.80%-9.57%, *p* < 0.05) (Fig 2).

#### Subgroup analysis of countries.

Firstly, studies spanning multiple countries were disaggregated into individual units for analysis. A total of 35 studies were ultimately included (Table 1 and S1 Fig). The prevalence of HVs in Indonesia, Singapore, Thailand, Cambodia, Malaysia, Vietnam were 17.49% (95%CI, 8.90%-31.51%, *p* < 0.01), 10.53% (95%CI, 0.56%-70.97%, *p* < 0.01), 4.36% (95%CI, 2.22%-8.42%, *p* < 0.01), 5.50% (95%CI, 2.39%-12.16%, *p* < 0.01), 4.32% (95%CI, 1.06%-15.97%, *p* < 0.01), 4.25% (95%CI, 2.11%-8.36%, *p* < 0.01), respectively. Only one study was identified for Laos and the Philippines, and the prevalence in Myanmar was not statistically significant (*p* > 0.05). Significant differences in prevalence of HVs were observed between countries (*p* < 0.05) (S1 Fig).

**Table 1:** Prevalence of Hantavirus in small mammals in different subgroups analysis.

Subgroup	Variables	No. studies	No. samples	No. positive	Prevalence (95%CI)	Heterogeneity		
						τ^2^value	I^2^/%	*p*-value
Countries	Thailand	7	2820	103	0.05(0.02,0.08)	0.797	91.7	<0.001
	Singaporean	2	2245	316	0.11(0.01,0.71)	4.770	99.6	<0.001
	Indonesia	7	1505	214	0.17(0.09,0.32)	0.946	95.3	<0.001
	Malaysia	4	308	20	0.04(0.00,0.16)	1.321	74.8	0.008
	Cambodian	2	1252	75	0.05(0.02,0.12)	0.357	91.1	0.008
	Vietnam	8	2659	139	0.04(0.02,0.08)	0.786	80.3	<0.001
	Laos	1	397	13	0.03(0.02,0.06)	–	–	–
	Philippine	1	376	1	0.00(0.00,0.01)	–	–	–
	Myanmar	3	244	5	0.03(0.01,0.06)	<0.001	0.0	0.419
Detection methods	IFA^a^	7	3295	215	0.09(0.05,0.17)	0.861	96.8	<0.001
	ELISA^b^	8	3502	553	0.10(0.04,0.25)	2.082	98.3	<0.001
	RT-PCR^c^	9	2584	68	0.03(0.01,0.06)	1.835	92.7	<0.001
Species	Bat	4	984	11	0.01(0.00,0.03)	0.583	52.4	0.098
	Shrews	5	399	33	0.04(0.01,0.13)	1.147	63.2	0.028
	Rodent	23	10423	942	0.08(0.05,0.13)	1.389	97.9	<0.001
Year of publication	1980-1999	4	1249	148	0.12(0.05,0.28)	1.062	96.9	<0.001
	2000-2009	7	3008	155	0.05(0.03,0.09)	0.621	90.0	<0.001
	2010-2019	12	5989	256	0.04(0.02,0.07)	1.2432	93.6	<0.001
	2020-2025	5	1560	447	0.11(0.02,0.43)	3.907	92.0	<0.001
Sample size	1-99	5	359	61	0.09(0.02,0.32)	2.779	88.4	<0.001
	100-499	15	3624	283	0.06(0.03,0.11)	1.703	92.6	<0.001
	500 or more	8	7823	649	0.05(0.02,0.10)	1.109	99.2	<0.001

^a^Indirect Immunofluorescence assay. ^b^Enzyme-Linked Immunosorbent assay.

^c^Reverse Transcription-Polymerase Chain Reaction.

#### Subgroup analysis of detection methods.

Firstly, studies employing multiple detection methods were disaggregated into individual units for analysis. A total of 30 studies were ultimately included (Table 1 and S2 Fig). The 24 studies were divided into three groups based on the detection methods used: IFA, ELISA, RT-PCR. The remaining six studies that used cannot distinguish methods or other methods were excluded, and a subgroup analysis was then performed. The prevalence for IFA, ELISA, RT-PCR were 9.35% (95%CI, 4.85%-17.26%, *p* < 0.01), 10.68% (95%CI, 4.05%-25.33%, *p* < 0.01), and 2.29% (95%CI, 0.87%-5.91%, *p* < 0.01), respectively, with ELISA showing the highest rate. A marginal p-value (*p* < 0.05) was reported among different detection methods (S2 Fig).

#### Subgroup analysis of species.

Firstly, studies encompassing multiple species were disaggregated into individual units for analysis. A total of 33 studies were ultimately included (Table 1 and S3 Fig). The prevalence of HVs in rodents, shrews, bats were 8.41% (95%CI, 5.35%-12.99%, *p* < 0.05), 3.90% (95%CI, 1.09%-13.05%, *p* < 0.05), 1.64% (95%CI, 0.41%-3.16%, *p* > 0.05), respectively. Significant differences in prevalence of HVs were observed between species (*p* < 0.05) (S3 Fig).

#### Subgroup analysis of publication years.

Subgroup analysis by publication year included a total of 28 studies (Table 1 and S4 Fig). The prevalence of HVs in 1980–1999, 2000–2009, 2010–2019, and 2020–2025 group were 11.99% (95%CI, 4.64%-27.63%, *p* < 0.05), 5.08% (95%CI, 2.79%-9.07%, *p* < 0.05), 3.86% (95%CI, 1.97%-7.45%, *p* < 0.05), 11.32% (95%CI, 2.07%-43.48%, *p* < 0.05), respectively. Not significant differences in prevalence of HVs were observed between publication years (*p* > 0.05) (S4 Fig).

#### Subgroup analysis of year of Sample size.

Subgroup analysis was divided into three groups based on sample size: small sample group (<100), medium sample group (100–500), and large sample group (>500) (Table 1 and S5 Fig). The prevalence of HVs in the small sample group, medium sample, large group were 9.02% (95%CI, 1.97%-32.87%, *p* < 0.05), 5.78% (95%CI, 2.96%-10.98%, *p* < 0.05), 5.00% (95%CI, 2.45%-9.94%, *p* < 0.05), respectively. Not significant differences in prevalence of HVs were observed between sample sizes (*p* > 0.05) (S5 Fig).

### Sensitivity analysis and publication bias

**Fig 3:**
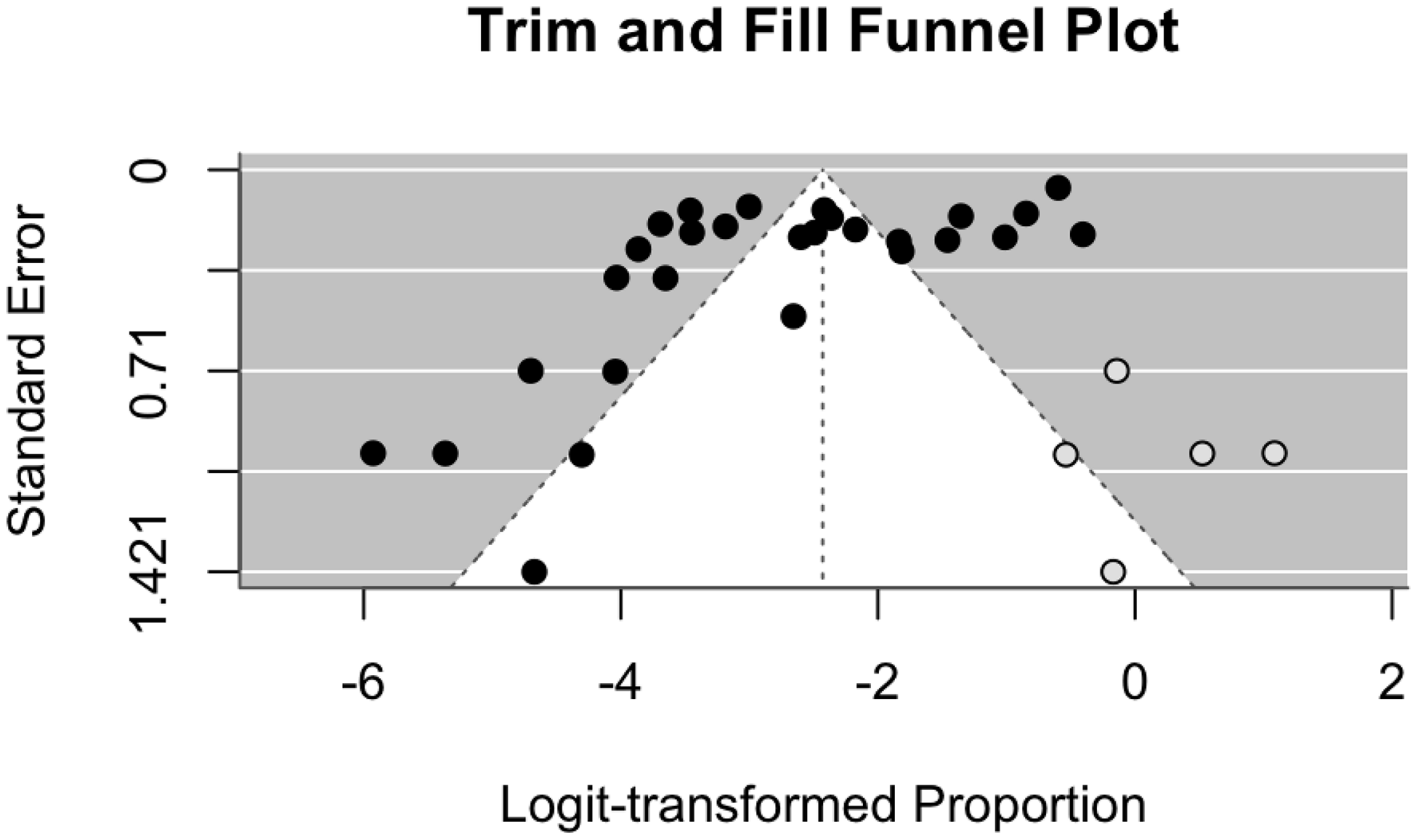
Trim and fill funnel plot of the prevalence of Hantavirus in small mammals in Southeast Asia.

The findings were reliable as the exclusion of any single study did not greatly change the overall results. Tests for publication bias gave mixed results: Egger’s test suggested possible bias (p < 0.001), but Begg’s test did not (*p* = 0.8911). The funnel plot was visually asymmetric (S6 Fig), and imply possible bias or differences between studies. The funnel plot generated by the trim-and-fill method estimated five missing studies (Fig 3). The adjusted effect increased by 11% (from -2.7392 to -2.4298) but stayed within an acceptable range after adding these. The corrected effect size remained statistically significant, demonstrating that the findings are robust despite potential existence of publication bias.

## Discussion

This meta-analysis indicated that the prevalence of HVs in small mammals in Southeast Asia (6.07%) is marginally higher than that reported in China (2.58–6.39%) [9,42]. The transmission of HVs is primarily related to the population of small animal hosts. And the population is closely associated with the hosts’ reproductive capacity, living environment, and climatic conditions. Southeast Asia is recognized as the origin of rodents, with more that 35 species of rodents in this region [26]. The temperature and humidity is high in this region, it could create favorable conditions for the survival and reproduction of small mammals. It leads to an increase in the population of small mammals, thereby the high prevalence of HVs [43].

**Fig 4:**
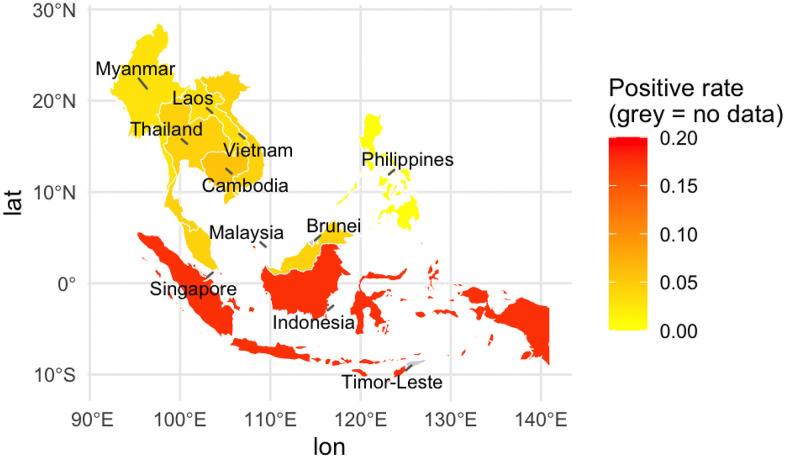
Geographic distribution heatmap of prevalence of Hantavirus in small animals in Southeast Asia. All geographic data processing and visualization were performed with R software (version 4.5.0), using base map data from Natural Earth (public domain, https://www.naturalearthdata.com/) retrieved via the rnaturalearth package.

According to the results of subgroup analysis by country, significant differences were observed in the prevalence of HVs in small mammals. Indonesia and Singapore have higher prevalence, while other countries have showed lower values. Indonesia has large areas of farmland and a suitable climate, while Singapore is densely populated and generates substantial waste [39]. Both of these factors provide ideal habitats for small mammals and contribute to a higher prevalence of HVs. This elevated prevalence poses a direct international risk, as highlighted by a confirmed case of human HVs infection imported from Indonesia to Germany [44]. The absence of positive cases in the Philippines may be related to factors such as the limited number of studies and small sample size. A geographical distribution heat map analysis showed that the prevalence of HVs in small mammals in low-latitude countries (Singapore, Indonesia) is significantly higher than in other Southeast Asian countries (Fig 4).The warm and humid climate of low-latitude regions facilitates small mammal reproduction, leading to higher population densities and enhanced survival of HVs [43]. Chinese research has found that small mammal populations tend to be larger in low-latitude regions [45]. However, other studies have shown that habitat quality has a greater impact on population size than latitude [46]. We suggest that future studies should explore the mechanism by which latitude affects the prevalence of HVs in small mammals.

Both IFA and ELISA detection methods showed higher prevalence than RT-PCR, which is consistent with Obando-Rico et al. [47]. The reason for this result may be that IFA and ELISA detect antigens or antibodies, thereby reflecting prior exposure of the virus and a relative higher prevalence. While RT-PCR detects the current HVs infection in small mammals.

This study found a significant difference in the prevalence of HVs between rodents and shrews, with rates of 8.07% and 3.90%, respectively (*p* < 0.05), while no statistically significant was observed between the prevalence of bat and rodents, and of bats and shrews (*p* > 0.05). A total of 10,423 rodents were included in this study, far exceeding the sample size of bats and shrews.

To further explore the species-specificity of HVs transmission, we conducted a more in-depth subgroup analysis. The results indicate that *R. norvegicus*, *Bandicota indica*, *R. rattus*, and *Suncus murinus* are the primary vectors for HVs transmission in Southeast Asia, with their respective being 14.01% (95%CI, 9.28%–20.61%, *p* < 0.01), 10.42% (95%CI, 9.28%–20.61%, *p* < 0.01), 4.01% (95%CI, 9.28%–20.61%, *p* < 0.01), and 3.90% (95%CI, 9.28%–20.61%, *p* < 0.05) (S7 Fig). Among these species, *R. norvegicus* exhibited the highest prevalence of HVs, which can be attributed to its status as a dominant species with a broad distribution spanning from tropical to arctic climates [39,48]. Their high adaptability facilitates the transmission of HVs.

In Southeast Asia, distinct HVs seropositivity patterns were observed across small mammal species: *B. indica*, *R. norvegicus*, *R. exulans*, *R. rattus*, and *S. murinus* are widely distributed and show relatively high prevalence of HVs [39]. In contrast, *R. argentiventer*, *R. tiomanicus*, and *Hipposideros* spp. have restricted distributions, and their prevalence of HVs may display regional fluctuations driven by habitat fragmentation and host specificity (S8 Fig). Notably, *B. indica*, *R. rattus*, and *S. murinus* are highly abundant in particular geographic regions. Yet, due to inadequate sampling efforts and constraints in research priorities, these species remain poorly investigated in current literature, potentially masking their actual role in HVs epidemiology.

Notably, the HVs data of bats should not be directly pooled with that of rodents or shrews for comparative analysis, given the substantial disparities in research contexts. Unlike the aforementioned small mammals, for which both molecular (RT-PCR) and serological (IFA/ELISA) data are available, current prevalence of HVs in bats is limited to genome detection by RT-PCR alone, and lacked to serological validation (e.g., antibody detection). The number of studies focusing on bats is significantly smaller than that on rodents, resulting in insufficient research coverage and limited data support. Therefore, the absence of statistical significance in the prevalence of HVs in bats observed in this study more likely underestimated the limitations of current research rather than reflects a true absence of epidemiological risk. The primary reasons for this finding are the insufficient number of studies on bats, coupled with a severe scarcity of serological data [3,33,35,36]. As bats are recognized reservoirs for numerous viruses, their potential role in transmission should not be underestimated. Future research must be strengthened to clarify their specific role in the ecology of HVs.

The marked heterogeneity in prevalence of HVs in different host species underestimates the critical role of host species in HVs transmission dynamics. Future research should prioritize investigations into HVs transmission mechanisms, as well as host ecology and behavior, to inform the development of targeted intervention strategies. The integration of geographic information systems (GIS) and molecular epidemiological approaches may help clarify the relationships between host species distributions and HVs transmission risk.

In this study, we conducted the first meta-analysis of the combined prevalence of HVs in small mammals in Southeast Asia. Heterogeneity testing revealed high heterogeneity (I² = 95.6%), prompting a subgroup analysis to explore the sources of heterogeneity. Subgroup analysis revealed significant differences in prevalence of HVs across countries and species (*p* < 0.05), while detection methods showed marginal significance (*p* = 0.051). Although the difference in prevalence of HVs by methods did not reach the traditional statistical significance threshold, the proximity to the critical value of 0.05 may suggest a potential influence of detection methods on the results. The marginal significance may reflect inadequate subgroup sample sizes, compromising statistical power and obscuring actual intergroup differences. The potential influence of detection methods requires further validation through larger sample sizes or standardized experimental designs.

Although this study is the first to conduct a meta-analysis on prevalence of HVs in Southeast Asia, several limitations should be noted: (1) We only searched English studies and missed non-English and grey literature, which may have left out important data. (2) Nine Southeast Asian countries were included in our study, however, some had very few studies or small samples. This uneven coverage could affect the generalizability of our results to the entire region. (3) This meta-analysis revealed significant heterogeneity among the studies. (4) The study detected significant publication bias (*p* < 0.001), which may affect the estimation of the overall positive rate. (5) Although different detection methods can affect the results of prevalence of HVs, the subgroup analysis by country in this study did not distinguish between these methods. (6) Given the high diversity of HVs subtypes and the complexity of viral strains carried by hosts (e.g., rodents), methods in this study poses certain challenges for the simultaneous differentiation and identification of multiple HVs subtypes—particularly in the overlapping regions of the Indian Ocean periphery and Southeast Asia, where viral strain coexistence is highly prevalent [49].

A significant result of Egger’s test and the asymmetry was observed in the funnel plot suggest the possibility of publication bias in the included literatures. Publication bias typically arises when studies with statistically significant or ‘positive’ findings (e.g., higher prevalence rates) are more likely to be published than those with non-significant or ‘negative’ results. This could imply that our original pooled prevalence estimates of 6.07% may be slightly overestimated. However, the trim-and-fill analysis indicated that any potential overestimation was relatively limited in magnitude, and the overarching conclusion—that HVs are present in small mammals across Southeast Asia—remained robust following adjustment. Nevertheless, readers are advised to interpret the absolute prevalence with this potential limitation in consideration.

To address current knowledge gaps, we recommend: (1) enhanced longitudinal surveillance and molecular epidemiological studies in under-represented regions (e.g., Cambodia, Laos) and key host species, particularly in highly endemic areas; (2) implementation of multicenter longitudinal studies incorporating molecular characterization to improve predictive accuracy and intervention efficacy. Research priorities should emphasize HVs ecology, spatiotemporal distribution patterns, and determinants of host population dynamics to inform evidence-based control strategies.

## Supporting information

S1 Fig Subgroup Forest Map by Country. (TIF)

S2 Fig Subgroup Forest Plot by Methods. (TIF)

S3 Fig Subgroup Forest Map by Specie. (TIF)

S4 Fig Subgroup Forest Map by Publication year. (TIF)

S5 Fig Subgroup Forest Map by Sample size. (TIF)

S6 Fig Publication bias funnel plot of the prevalence of Hantavirus carried by small mammals in Southeast Asia. (TIF)

S7 Fig Subgroup Forest Map by Specie and Genu. Complete detection data were available for 1,093 rodents, but species-specific sample sizes were not reported according to Griffiths et al. (2022). Species-level prevalence was estimated using overall detection rates and the species distribution among 1,126 rodents (990 *Rattus norvegicu*, 136 *Rattus rattus*).
(TIF)

S8 Fig Heat map showing the prevalence of Hantavirus in different small mammals in Southeast Asia. *Bandicota indica* (A), *Rattus norvegicus* (B), *Rattus rattus* (C), *Rattus hosaensis* (D), *Suncus murinus* (E), *Rattus exulans* (F), *Rattus tiomanicus* (G), *Bandicota savilei* (H), *Mus caroli* (I), *Rattus argentiventer* (J), *Rattus losea* (K), *Mus cervicolor* (L), *Rattus tanezumi* (M), and *Hipposideros* bats (N). All geographic data processing and visualization were performed with R software (version 4.5.0), using base map data from Natural Earth (public domain, https://www.naturalearthdata.com/) retrieved via the rnaturalearth and rnaturalearthdata package.
(TIF)

S1 Table Characteristics of articles included in the Meta-analysis. ^a^ Indirect immunofluorescence assay. ^b^ Enzyme-Linked immunosorbent assay. ^c^ Reverse transcription-polymerase chain reaction. ^d^ Cross-Sectional Study. (DOC)

S2 Table Literature Quality Evaluation. (DOC)

S1 File PRISMA 2020 Checklist. From: Page MJ, McKenzie JE, Bossuyt PM, Boutron I, Hoffmann TC, Mulrow CD, et al. The PRISMA 2020 statement: an updated guideline for reporting systematic reviews. BMJ 2021;372:n71. https://doi.org/10.1136/bmj.n71. This work is licensed under CC BY 4.0. To view a copy of this license, visit https://creativecommons.org/licenses/by/4.0/deed.en.
(DOCX)
