# Supplementary material for: Studies on prevalence of Hantavirus in small mammals in Southeast Asia: A systematic review and meta-analysis
Source: PLoS Negl Trop Dis. 2026 Mar 12;20(3):e0014075. doi: 10.1371/journal.pntd.0014075 (PMC12981452; doi:10.1371/journal.pntd.0014075)
Supplement: S1 Table — (DOC) [file pntd.0014075.s009.doc]

**S1 Table. Characteristics of articles included in the Meta-analysis**

| Author, year | Coutries^a^ | Species^b^ | Detection  methods^c^ | Total | No.  positive | Type | Quality  score |
| --- | --- | --- | --- | --- | --- | --- | --- |
| Elwell et al, 1985[1] | Thailand | Rodent | IFA^a^ | 288 | 22 | CSS^d^ | 6 |
| Tantivanich et al, 1992[2] | Thailand | Rodent(78), Shrews(28) | IFA | 106 | 20 | CSS | 9 |
| Ibrahim et al, 1996[3] | Indonesia | Rodent | IFA | 655 | 26 | CSS | 8 |
| Praseno et al, 1998[4] | Indonesia | Rodent | IFA | 200 | 60 | CSS | 7 |
| Nitatpattana et al, 2000[5] | Thailand | Rodent | ELISA^b^ | 680 | 21 | CSS | 8 |
| Lam et al, 2001[6] | Malaysia | Rodent | IFA | 131 | 18 | CSS | 8 |
| Nitatpattana et al, 2002[7] | Thailand | Rodent | ELISA | 632 | 13 | CSS | 9 |
| Reynes et al, 2003[8] | Cambodia | Rodent | ELISA | 660 | 54 | CSS | 8 |
| Hugot et al, 2006[9] | Thailand | Rodent | RT-PCR^c^ | 61 | 4 | CSS | 8 |
| Pattamadilok et al, 2006[10] | Thailand | Rodent | ELISA | 402 | 7 | CSS | 7 |
| Truong et al, 2009[11] | Vietnam | Rodent(401), Shrews(41) | Other | 442 | 38 | CSS | 7 |
| Johansson et al, 2010[12] | Singapore | Rodent | PCR | 1152 | 28 | CSS | 8 |
| Blasdell et al, 2011[13] | Cambodia(592), Laos(397), Thailand(651) | Rodent | IFA | 1640 | 50 | CSS | 9 |
| Kosasih et al, 2011[14] | Indonesia | Rodent | ELISA | 245 | 25 | CSS | 7 |
| Luan et al, 2012[15] | Vietnam | Rodent(1066), Shrews(245) | Other | 1311 | 62 | CSS | 8 |
| Ibrahim et al, 2013[16] | Indonesia | Rodent | ELISA（59）, Other（170） | 229 | 47 | CSS | 9 |
| Koma et al, 2013[17] | Vietnam | Rodent | ELISA | 100 | 14 | CSS | 9 |
| Nguyen Van et al, 2015[18] | Vietnam | Rodent | IFA | 275 | 19 | CSS | 9 |
| Arai et al, 2016[19] | Philippines | Bat | RT-PCR | 376 | 1 | CSS | 7 |
| Hamdan et al, 2017[20] | Malaysia | Rodent | ELISA | 53 | 0 | CSS | 8 |
| Arai et al, 2019[21] | Vietnam | Bat | RT-PCR | 215 | 1 | CSS | 8 |
| Arai et al, 2019[22] | Myanmar(121), Vietnam(156) | Bat | RT-PCR | 277 | 7 | CSS | 8 |
| Zana et al, 2019[23] | Malaysia | Bat | RT-PCR | 116 | 2 | CSS | 8 |
| Kikuchi et al, 2020[24] | Indonesia(6), Malaysia(8), Myanmar(25), Vietnam(36) | Shrews | RT-PCR | 75 | 1 | CSS | 9 |
| Kikuchi et al, 2021[25] | Vietnam(124), Myanmar(98) | Rodent | RT-PCR | 222 | 2 | CSS | 9 |
| Griffiths et al, 2022[26] | Singapore | Rodent | ELISA | 1093 | 388 | CSS | 9 |
| Susanti et al, 2022[27] | Indonesia | Rodent | ELISA | 80 | 32 | CSS | 9 |
| Miura et al, 2025[28] | Indonesia | Rodent(80), Shrews(10) | RT-PCR | 90 | 24 | CSS | 8 |

^a^ Numbers in parentheses indicate sample size from each country.

^b^ Numbers in parentheses indicate sample size for each species.

^c^ Numbers in parentheses indicate sample size detected by each method.

^d^ Cross-Sectional Study.

**References**

1. Elwell MR, Ward GS, Tingpalapong M, LeDuc JW. Serologic evidence of hantaan-like virus in rodents and man in Thailand. Southeast Asian Journal of Tropical Medicine and Public Health. 1985;16(3):349-54.

2. Tantivanich S, Ayuthaya PI, Usawattanakul W, Imphand P. Hantaanvirus among urban rats from a slum area in Bangkok. The Southeast Asian journal of tropical medicine and public health. 1992;23(3):504-9.

3. Ibrahim IN, Sudomo M, Morita C, Uemura S, Muramatsu Y, Ueno H, et al. Seroepidemiological survey of wild rats for Seoul virus in Indonesia. Japanese Journal of Medical Science and Biology. 1996;49(2):69-74.

4. Praseno, Suwarso. Isolation and presumptive serological characterization of hantavirus from wild rat (Bandicota indica). Medical Journal of Indonesia. 1998;7(3):115-8.

5. Nitatpattana N, Chauvancy G, Dardaine J, Poblap T, Jumronsawat K, Tangkanakul W, et al. Serological study of hantavirus in the rodent population of Nakhon Pathom and Nakhon Ratchasima Provinces Thailand. The Southeast Asian journal of tropical medicine and public health. 2000;31(2):277-82.

6. Lam SK, Chua KB, Myshrall T, Devi S, Zainal D, Afifi SA, et al. Serological evidence of hantavirus infections in Malaysia. The Southeast Asian journal of tropical medicine and public health. 2001;32(4):809-13.

7. Nitatpattana N, Henrich T, Palabodeewat S, Tangkanakul W, Poonsuksombat D, Chauvancy G, et al. Hantaan virus antibody prevalence in rodent populations of several provinces of northeastern Thailand. Tropical Medicine and International Health. 2002;7(10):840-5.

8. Reynes JM, Soares JL, Hüe T, Bouloy M, Sun S, Kruy SL, et al. Evidence of the presence of Seoul virus in Cambodia. Microbes Infect. 2003;5(9):769-73.

9. Hugot JP, Plyusnina A, Herbreteau V, Nemirov K, Laakkonen J, Lundkvist Å, et al. Genetic analysis of Thailand hantavirus in Bandicota indica trapped in Thailand. Virology Journal. 2006;3:72.

10. Pattamadilok S, Lee BH, Kumperasart S, Yoshimatsu K, Okumura M, Nakamura I, et al. Geographical distribution of hantaviruses in Thailand and potential human health significance of Thailand virus. American Journal of Tropical Medicine and Hygiene. 2006;75(5):994-1002.

11. Truong TT, Yoshimatsu K, Araki K, Lee BH, Nakamura I, Endo R, et al. Molecular epidemiological and serological studies of hantavirus infection in Northern Vietnam. Journal of Veterinary Medical Science. 2009;71(10):1357-63.

12. Johansson P, Yap G, Low HT, Siew CC, Kek R, Ng LC, et al. Molecular characterization of two hantavirus strains from different rattus species in Singapore. Virology Journal. 2010;7:15.

13. Blasdell K, Cosson JF, Chaval Y, Herbreteau V, Douangboupha B, Jittapalapong S, et al. Rodent-borne hantaviruses in Cambodia, Lao PDR, and Thailand. EcoHealth. 2011;8(4):432-43.

14. Kosasih H, Ibrahim IN, Wicaksana R, Alisjahbana B, Hoo Y, Yo IH, et al. Evidence of human hantavirus infection and zoonotic investigation of hantavirus prevalence in rodents in Western Java, Indonesia. Vector-Borne and Zoonotic Diseases. 2011;11(6):709-13.

15. Luan VD, Yoshimatsu K, Endo R, Taruishi M, Huong VT, Dat DT, et al. Studies on hantavirus infection in small mammals captured in southern and central highland area of Vietnam. J Vet Med Sci. 2012;74(9):1155-62.

16. Ibrahim I, Shimizu K, Yoshimatsu K, Yunianto A, Salwati E, Yasuda SP, et al. Epidemiology of hantavirus infection in Thousand Islands regency of Jakarta, Indonesia. 2013;75(8): 1003-8.

17. Koma T, Yoshimatsu K, Yasuda SP, Li T, Amada T, Shimizu K, et al. A survey of rodent-borne pathogens carried by wild Rattus spp. in Northern Vietnam. Epidemiol Infect. 2013;141(9):1876-84.

18. Nguyen Van C, Carrique-Mas J, Hien Vo B, Nguyen Ngoc A, Ngo Tri T, Nguyet Lam A, et al. Rodents and Risk in the Mekong Delta of Vietnam: Seroprevalence of Selected Zoonotic Viruses in Rodents and Humans. Vector-Borne and Zoonotic Diseases. 2015;15(1):65-72.

19. Arai S, Taniguchi S, Aoki K, Yoshikawa Y, Kyuwa S, Tanaka-Taya K, et al. Molecular phylogeny of a genetically divergent hantavirus harbored by the Geoffroy's rousette (Rousettus amplexicaudatus), a frugivorous bat species in the Philippines. Infect Genet Evol. 2016;45:26-32.

20. Hamdan NE, Ng YL, Lee WB, Tan CS, Khan FA, Chong YL. Rodent Species Distribution and Hantavirus Seroprevalence in Residential and Forested areas of Sarawak, Malaysia. Trop Life Sci Res. 2017;28(1):151-9.

21. Arai S, Aoki K, Nguyen Tru'ong S, Vu'ong Tan T, Kikuchi F, Kinoshita G, et al. Dakrong virus, a novel mobatvirus (Hantaviridae) harbored by the Stoliczka's Asian trident bat (Aselliscus stoliczkanus) in Vietnam. Scientific Reports. 2019; 9(1):10239.

22. Arai S, Kikuchi F, Bawm S, Nguyen Truong S, Lin KS, Vuong Tan T, et al. Molecular Phylogeny of Mobatviruses (Hantaviridae) in Myanmar and Vietnam. Viruses-Basel. 2019;11(3):228.

23. Zana B, Kemenesi G, Buzás D, Csorba G, Görföl T, Khan FAA, et al. Molecular Identification of a Novel Hantavirus in Malaysian Bronze Tube-Nosed Bats (Murina aenea). Viruses. 2019;11(10):887.

24. Kikuchi F, Aoki K, Ohdachi SD, Tsuchiya K, Motokawa M, Jogahara T, et al. Genetic Diversity and Phylogeography of Thottapalayam thottimvirus (Hantaviridae) in Asian House Shrew (Suncus murinus) in Eurasia. Frontiers in Cellular and Infection Microbiology. 2020;10:438.

25. Kikuchi F, Senoo K, Arai S, Tsuchiya K, Nguyen Truong S, Motokawa M, et al. Rodent-Borne Orthohantaviruses in Vietnam, Madagascar and Japan. Viruses-Basel. 2021;13(7)1343.

26. Griffiths J, Yeo HL, Yap G, Mailepessov D, Johansson P, Low HT, et al. Survey of rodent-borne pathogens in Singapore reveals the circulation of Leptospira spp., Seoul hantavirus, and Rickettsia typhi. Scientific reports. 2022;12(1):2692.

27. Susanti HN, Noor SM, Dharmayanti N, Randusari P, Shimizu K, Hirayama K, et al. Serological Surveillance of Zoonotic Pathogens in Rats in Markets in Bogor, Indonesia. Vector Borne Zoonotic Dis. 2022;22(11):568-70.

28. Miura K, Chambers J, Takahashi N, Nuradji H, Dharmayanti NI, Randusari P, et al. Coinfection with Orthohantavirus and Leptospira spp. in Rats Collected from Markets in Indonesia. Vector-Borne and Zoonotic Diseases. 2025;25(1):43-8.
